# Growth and Physiological Responses of Magnoliaceae to NaCl Stress

**DOI:** 10.3390/plants13020170

**Published:** 2024-01-08

**Authors:** Xiuting Zhao, Ling Tian, Zhonglong Zhu, Ziyang Sang, Lvyi Ma, Zhongkui Jia

**Affiliations:** 1 Key Laboratory Silviculture and Conservation of MOE, College of Forestry, Beijing Forestry University, Beijing 100083, China; zhaoxt0906@163.com (X.Z.); 14760025278@163.com (L.T.); thefuturehero@163.com (Z.Z.); maluyi@bjfu.edu.cn (L.M.); 2 Forest Science Research Institute of Wufeng Tujia Autonomous County, Wufeng 443400, China; sangziyang@21cn.com; 3 *Magnolia wufengensis* Research Center, Beijing Forestry University, Beijing 100083, China; 4 National Energy R&D Center for Non-Food Biomass, Beijing Forestry University, Beijing 100083, China

**Keywords:** *Magnolia wufengensis*, salt stress, growth, antioxidation, osmotic adjustment

## Abstract

The growth and physiological characteristics of four Magnoliaceae plants (*Yulania biondii*, *Yulania denudata*, and two varieties of *Magnolia wufengensis* (Jiaohong 1 and Jiaohong 2)) were investigated. Four Magnoliaceae plants were subjected to various concentrations of NaCl for 60 days: 0 mM, 60 mM, 120 mM, 180 mM, and 240 mM. The leaf water content (LWC), relative growth rate of plant height and stem diameter, photosynthetic pigments, and photosynthetic rate (*P_n_*) decreased during the NaCl treatments, indicating slowed growth and photosynthesis. Malondialdehyde (MDA), Na^+^, superoxide dismutase (SOD) activity, peroxidase (POD) activity, ascorbic acid (AsA) content, and soluble sugar content all increased while K^+^ decreased. Ascorbate peroxidase (APX) activity, glutathione (GSH), soluble protein, and proline first increased after decreasing with increasing NaCl concentration. Principal component 1 (PC1) had larger loading values for growth and photosynthesis indices, while principal component 2 (PC2) exhibited larger loading values for antioxidant substances and osmotic adjustment substances; the correlation analysis showed that PC1 and PC2 had negative correlations. The four Magnoliaceae plants exhibited largely variable growth and physiological activities in response to NaCl. *Yulania denudata* exhibited greater reductions in growth and photosynthesis and greater decreases in antioxidant enzyme activities and osmotic adjustment substances, which indicated poor tolerance to salt stress. Among the four Magnoliaceae plants, Jiaohong 1 exhibited the greatest salt tolerance, followed by Jiaohong 2, *Yulania biondii*, and *Yulania denudata*.

## 1. Introduction

Soil salinity is one of the most severe abiotic stresses that plants suffer worldwide. It can have a significant impact on plant growth and soil productivity and is a major factor contributing to ecological degradation. Over 8.33 × 108 hm^2^, or approximately 8.7% of the earth’s land area, comprises saline-alkali areas on a global scale [1]. In China, saline-alkali land covers 7.67 × 106 hm^2^ and is expanding [1].

Low water potential in saline soils results in water loss from plant roots, cell shrinkage, slowed plant growth, and reductions in plant height, leaf count, stem diameter, water content, and dry weight [2]. The stomata close as a consequence of a shortage of water, limiting stomata-dependent photosynthesis and lowering the net photosynthetic rate. Furthermore, salt-induced ion toxicity damages the photosynthetic reaction center, overloads electron absorption, results in the production of reactive oxygen species (ROS), and eventually leads to oxidative damage [3].

ROS include singlet oxygen (^1^O_2_^−^), hydrogen peroxide (H_2_O_2_), hydroxyl radicals (OH), superoxide anions (O_2_^•−^), and so on [4,5,6]. ROS can result in cell damage and death by oxidatively harming proteins, DNA, and RNA, as well as membranes (through membrane lipid peroxidation) [7]. Plants bolster their antioxidant systems to maintain a balance of ROS through the use of two categories of antioxidants [8,9]. Superoxide dismutase (SOD), peroxidase (POD), catalase (CAT), ascorbate peroxidase (APX), monodehydroascorbate reductase (MDHAR), dehydroascorbate reductase (DHAR), and glutathione reductase (GR) are varieties of enzymatic antioxidants [10]. Ascorbic acid (AsA), glutathione (GSH), carotenoids, tocopherols, flavonoids, and other nonenzymatic antioxidants are among the other categories of antioxidants [11]. SOD catalyzes O_2_^•−^ to H_2_O_2_ and O_2_. POD and CAT decompose hydrogen peroxide into O_2_ and H_2_O [8]. The AsA–GSH cycle, which comprises AsA, GSH, APX, MDHAR, DHAR, and GR, has been shown to play a positive role in many plants that are exposed to salt stress [3,12]. AsA can remove H_2_O_2_ effectively, which not only eliminates excess light energy used in photosynthesis but also maintains the activity of antioxidant enzymes [7]. GSH functions as an antioxidant and regulates a number of metabolic processes [12]. The cycle consists of AsA and GSH with the assistance of APX, MDHAR, DHAR, and GR. The key antioxidant enzyme involved in removing H_2_O_2_ is APX, which catalyzes the oxidation of ascorbic acid in the first step of the AsA–GSH cycle [13]. GR reduces oxidized glutathione, allowing the chain reaction to complete and continue [7].

Ions and organic solutes enable plants to maintain osmotic balance. To maintain equilibrium under osmotic pressure, large amounts of Na^+^ and Cl^−^ enter root cells, which may disturb ion absorption because elevated concentrations of Na^+^ impact the absorption of K^+^. Furthermore, it may result in ionic toxicity, which breaks down protein and biofilm structures and disrupts osmotic pressure and other biological processes [14]. According to previous studies, the organic solutes proline, soluble sugar, and glycine betaine maintain a steady osmotic pressure [6,15,16,17]. These substances can stabilize protein structures and act as osmotic agents [18,19,20]. The production of these organic osmotic protectants requires much more energy than the production of ions, which may impede plant growth [14].

Magnoliaceae is an original family distributed in the tropical and subtropical regions of Asia and America [21]. The Magnoliaceae family contains approximately 246 species worldwide, and China contains the greatest number of species. A new Magnoliaceae species, *Magnolia wufengensis*, was found in Wufeng County, Hubei Province, China, in 2004 [22,23]. Due to its tall columnar stems and large red blossoms, *M. wufengensis* is a superb decorative tree species and a greening species with high commercial potential [21]. *M. wufengensis* has been introduced and cultivated in many regions. However, the plants exhibited poor growth when introduced to saline areas. Despite these findings, no research has compared *M. wufengensis* to other Magnoliaceae plants or evaluated its tolerance to salt. In the present study, the growth, photosynthesis, antioxidant, and osmoregulatory indicators of four Magnoliaceae plants were determined at various salt concentrations to explore the effects of salt stress on their growth and physiology and to fully evaluate their salt tolerance. These findings provide guidance for the popularization and cultivation of Magnoliaceae in saline soils and shed light on the physiological mechanisms of salt tolerance.

## 2. Results

### 2.1. Effects of NaCl on Plant Growth

**Figure 1:**
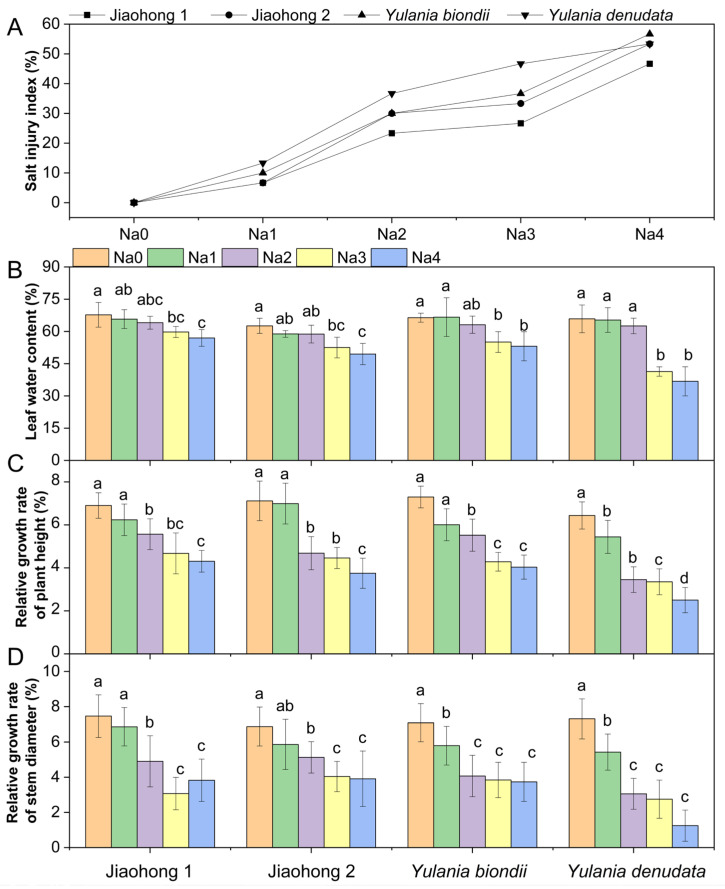
Effects of NaCl on the growth parameters of the four Magnoliaceae plants. (**A**) Salt injury index (SI); (**B**) Leaf water content (LWC); (**C**) Relative growth rate of plant height; (**D**) Relative growth rate of stem diameter. Na0: 0 mM NaCl; Na1: 60 mM NaCl; Na2: 120 mM NaCl; Na3: 180 mM NaCl; and Na4: 240 mM NaCl. Treatments for a given species were compared. Different lowercase letters in the figure indicate significant differences (*p* < 0.05 by Duncan’s test) among treatments. The bars indicate the standard deviation.

The salt injury index can visually indicate the degree of salt damage. In this experiment, salt stress began to develop at 60 mM and above (Figure 1A). The salt injury index increased with increasing NaCl concentration, indicating that salt stress was more severe. The salt injury index of Jiaohong 1 was the lowest in the 60–240 mM NaCl treatments, whereas that of *Y. denudata* was the highest in the 60–180 mM NaCl treatments, at 13.33%, 36.67%, and 46.67%, respectively. With the 240 mM NaCl treatment, little difference was observed in the degree of salt damage between Jiaohong 2, *Y. biondii*, and *Y. denudata*. Leaf water content (LWC) was determined in the leaves of the four Magnoliaceae plants (Figure 1B). The LWC of the four Magnoliaceae plants decreased significantly (*p* < 0.05) in the 180–240 mM NaCl treatments, and that of *Y. denudata* decreased the most, at 41.34% and 36.79%, respectively.

With increasing NaCl concentrations, both the height and stem diameter decreased in all plants (Figure 1C,D). The relative growth rates of plant height and stem diameter differed. NaCl stress at 120 mM and above caused reduced plant height and stem diameter growth in all four Magnoliaceae plants (*p* < 0.05), and *Y. denudata* was the most severely affected plant. The relative growth rate of plant height decreased by 46.63–61.18%, and the relative growth rate of stem diameter decreased by 58.14–82.90% in the *Y. denudata* treated with 120–240 mM NaCl.

### 2.2. Effects of NaCl on Photosynthetic Pigment Content and Photosynthesis

**Figure 2:**
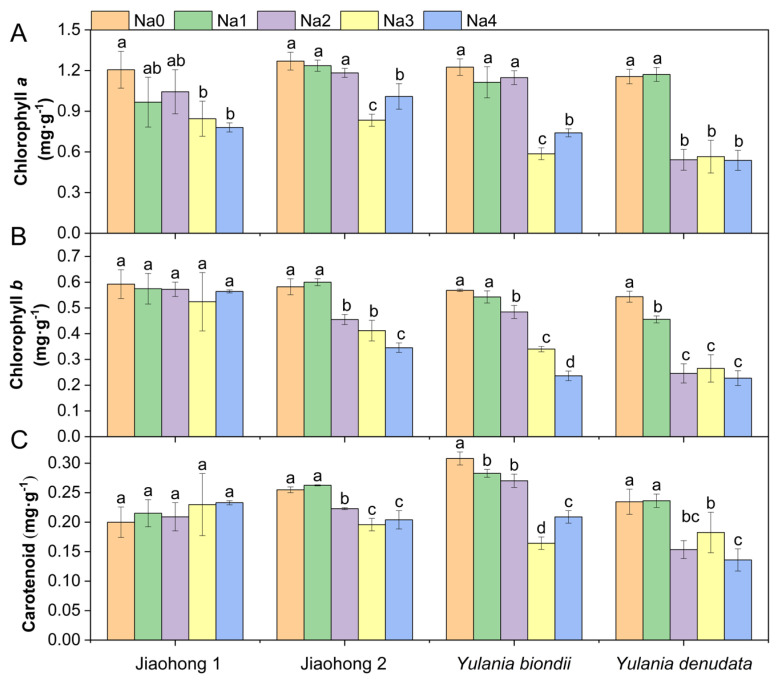
Effects of NaCl on the photosynthetic pigment contents of the four Magnoliaceae plants. (**A**) Chlorophyll *a*; (**B**) Chlorophyll *b*; (**C**) Carotenoid. Na0: 0 mM NaCl; Na1: 60 mM NaCl; Na2: 120 mM NaCl; Na3: 180 mM NaCl; and Na4: 240 mM NaCl. Treatments for a given species were compared. Different lowercase letters in the figure indicate significant differences (*p* < 0.05 by Duncan’s test) among treatments. The bars indicate the standard deviation.

The chlorophyll *a* content decreased after treatment with NaCl, whereas there were differences among the four Magnoliaceae plants (Figure 2A). The decreases in chlorophyll *a* in Jiaohong 1, Jiaohong 2, and *Y. biondii* were significant in the 180–240 mM NaCl treatments (*p* < 0.05) and significant in the 120–240 mM NaCl treatments in *Y. denudata*. The chlorophyll *b* and carotenoid contents of Jiaohong 1 did not change significantly under salt stress, but the contents of the other three Magnoliaceae plants decreased significantly (Figure 2B,C; *p* < 0.05).

**Figure 3:**
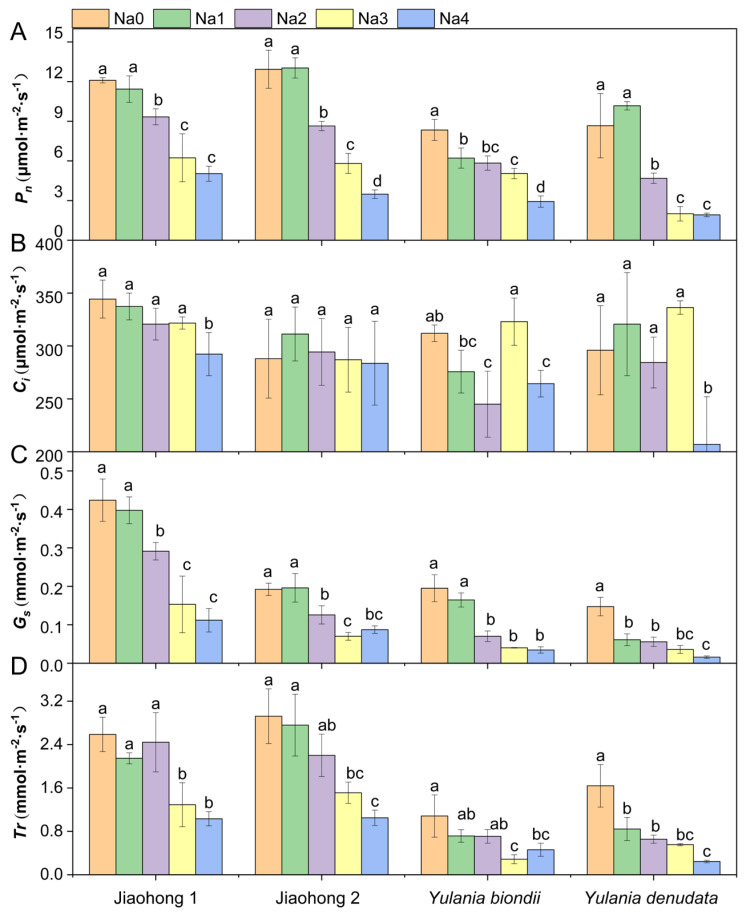
Effects of NaCl on the photosynthetic parameters of the four Magnoliaceae plants. (**A**) Net photosynthetic rate (*P_n_*); (**B**) Intercellular CO_2_ concentration (*C_i_*); (**C**) Stomatal conductance (*G_s_*); (**D**) Transpiration rate (*Tr*). Na0: 0 mM NaCl; Na1: 60 mM NaCl; Na2: 120 mM NaCl; Na3: 180 mM NaCl; and Na4: 240 mM NaCl. Treatments for a given species were compared. Different lowercase letters in the figure indicate significant differences (*p* < 0.05 by Duncan’s test) among treatments. The bars indicate the standard deviation.

Photosynthetic rate (*P_n_*) differed among the four Magnoliaceae plants under normal growth conditions (Figure 3A). The *P_n_* of *M. wufengensis* (Jiaohong 1 and Jiaohong 2) was greater than that of *Y. biondii* and *Y. denudata*, possibly due to species differences. Under 120–240 mM NaCl stress, the *P_n_* of all Magnoliaceae plants decreased significantly, and that of *Y. denudata* was the lowest, at 4.69 μmol·m^−2^·s^−1^, 2.00 μmol·m^−2^·s^−1^ and 1.92 μmol·m^−2^·s^−1^ (*p* < 0.05). Intercellular CO_2_ concentration (*C_i_*) did not significantly change in Jiaohong 1 or Jiaohong 2 under salt stress (Figure 3B; *p* > 0.05). *C_i_* in *Y. denudata* in the 240 mM NaCl treatment was only 207 μmol·m^−2^·s^−1^, which was significantly lower than that in the other treatments. Stomatal conductance (*G_s_*) tended to decrease as the NaCl concentration increased (Figure 3C). There was no significant difference in *G_s_* between the 60 mM NaCl treatment and the control in Jiaohong 1, Jiaohong 2, or *Y. biondii* (*p* > 0.05), whereas in *Y. denudata*, *G_s_* tended to significantly decrease (*p* < 0.05). Transpiration rate (*Tr*) for each species under different NaCl concentrations is shown in Figure 3D. There were no significant differences in *Tr* for Jiaohong 1, Jiaohong 2, or *Y. biondii* when exposed to 60–120 mM NaCl (*p* > 0.05), whereas the *Tr* of *Y. denudata* decreased significantly under 60 mM NaCl treatment and above (*p* < 0.05). Moreover, *Tr* in Jiaohong 1 and Jiaohong 2 was higher than that in *Y. biondii* and *Y. denudata* in all treatments.

### 2.3. Effects of NaCl on Antioxidant Enzymes, Nonenzymatic Antioxidants, and Membrane Lipid Peroxidation

**Figure 4:**
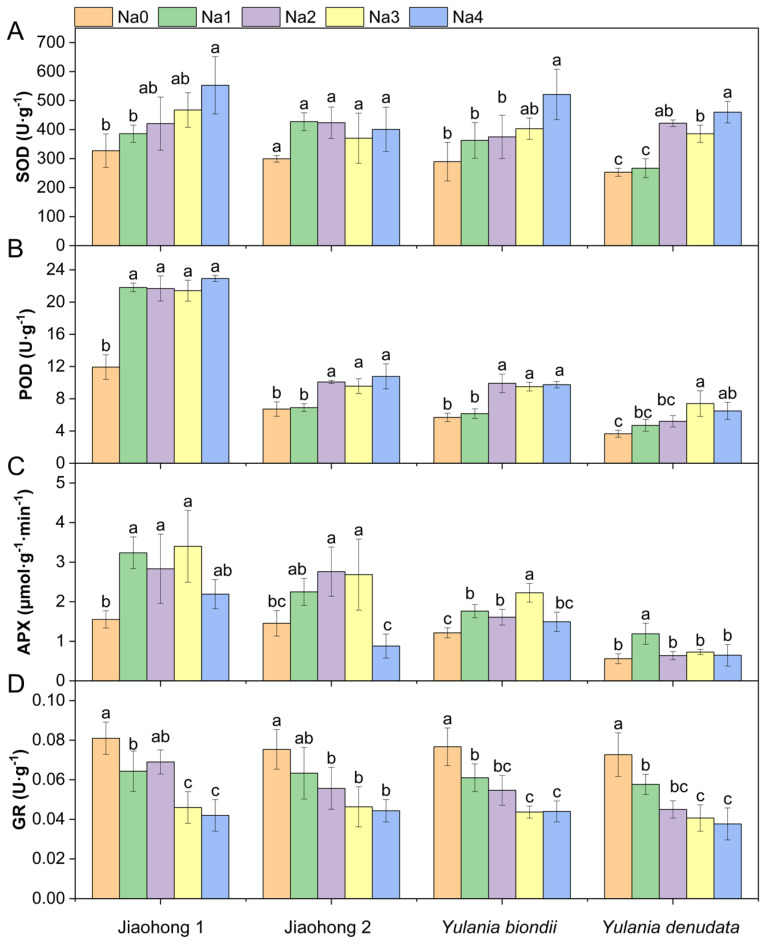
Effects of NaCl on the antioxidant enzyme activities of four Magnoliaceae plants. (**A**) Superoxide dismutase (SOD); (**B**) Peroxidase (POD); (**C**) Ascorbate peroxidase (APX); (**D**) Glutathione reductase (GR). Na0: 0 mM NaCl; Na1: 60 mM NaCl; Na2: 120 mM NaCl; Na3: 180 mM NaCl; and Na4: 240 mM NaCl. Treatments for a given species were compared. Different lowercase letters in the figure indicate significant differences (*p* < 0.05 by Duncan’s test) among treatments. The bars indicate the standard deviation.

Antioxidant enzyme activities in the leaves of the four Magnoliaceae plants are presented in Figure 4. The SOD and POD activities increased overall with increasing NaCl concentrations (Figure 4A,B). There were significant differences in SOD activity in Jiaohong 1, *Y. biondii*, and *Y. denudata* (*p* < 0.05), but these differences were not significant in Jiaohong 2 (*p* > 0.05). The increase in POD activity in Jiaohong 1 was most pronounced, increasing by approximately 80% in the NaCl treatment group. NaCl stress had an effect on APX in the four Magnoliaceae plants (Figure 4C). The APX activity of Jiaohong 1, Jiaohong 2, and *Y. biondii* significantly increased with 60–180 mM NaCl treatment (*p* < 0.05). In particular, APX activity was the highest in Jiaohong 1 in the 180 mM NaCl treatment, reaching 3.40 μmol·g^−1^·min^−1^. The GR activity of *Y. biondii* and *Y. denudata* decreased significantly under NaCl stress (Figure 4D; *p* < 0.05).

**Figure 5:**
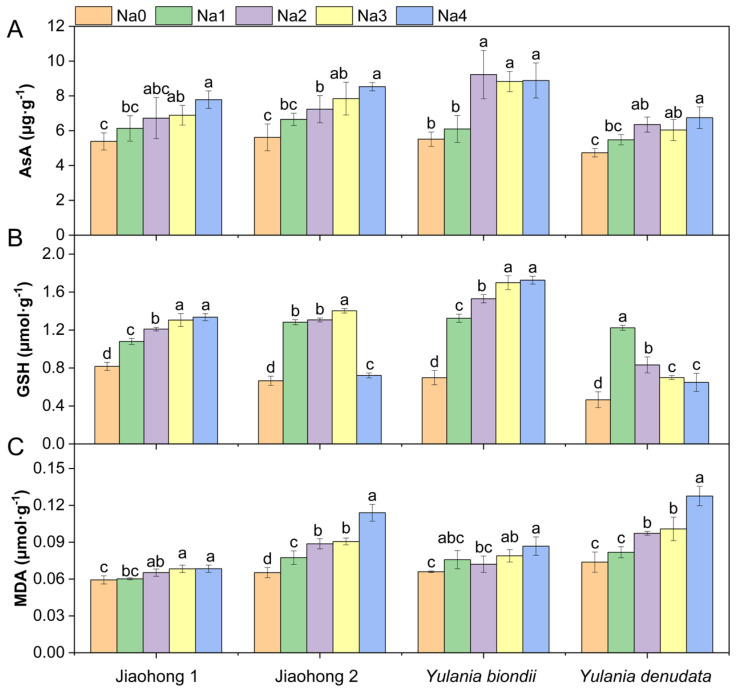
Effects of NaCl on the nonenzymatic antioxidants and membrane lipid peroxidation of the four Magnoliaceae plants. (**A**) Ascorbic acid (AsA); (**B**) Glutathione (GSH); (**C**) Malondialdehyde (MDA). Na0: 0 mM NaCl; Na1: 60 mM NaCl; Na2: 120 mM NaCl; Na3: 180 mM NaCl; and Na4: 240 mM NaCl. Treatments for a given species were compared. Different lowercase letters in the figure indicate significant differences (*p* < 0.05 by Duncan’s test) among treatments. The bars indicate the standard deviation.

An increasing AsA content was observed in all four Magnoliaceae plants under all NaCl treatments (Figure 5A). In particular, the AsA content increased by 67.03% in *Y. biondii* after 120 mM NaCl treatment. The AsA content of Jiaohong 1 was significantly different under 180–240 mM NaCl, whereas that of the other species was significantly different under 120–240 mM NaCl (*p* < 0.05). There were significant differences in the GSH content among the different NaCl concentrations (Figure 5B; *p* < 0.05). The GSH content of Jiaohong 2 and *Y. denudata* first increased after decreasing with increasing NaCl concentration. In contrast, Jiaohong 1 and *Y. biondii* increased continuously. The MDA content (Figure 5C) significantly increased with increasing NaCl stress (*p* < 0.05), whereas the MDA content in Jiaohong 2 and *Y. denudata* increased more sharply. The Malondialdehyde (MDA) content of *Y. denudata* cultivated in 240 mM NaCl was the highest of all treatments at 0.13 μmol·g^−1^.

### 2.4. Effects of NaCl on Osmotic Adjustment

**Figure 6:**
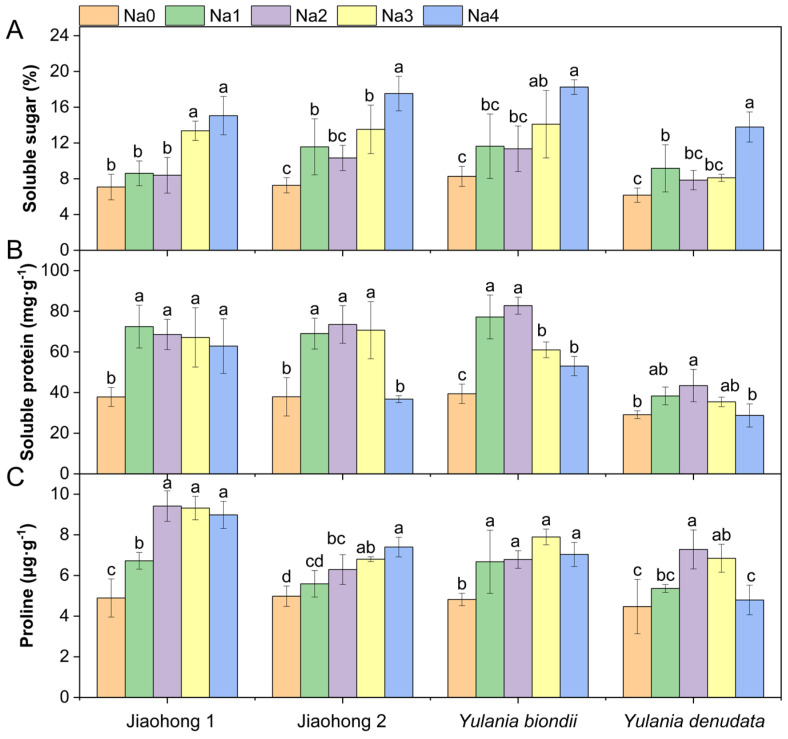
Effects of NaCl on the osmotic adjustment of the four Magnoliaceae plants. (**A**) Soluble sugar; (**B**) Soluble protein; (**C**) Proline. Na0: 0 mM NaCl; Na1: 60 mM NaCl; Na2: 120 mM NaCl; Na3: 180 mM NaCl; and Na4: 240 mM NaCl. Treatments for a given species were compared. Different lowercase letters in the figure indicate significant differences (*p* < 0.05 by Duncan’s test) among treatments. The bars indicate the standard deviation.

Salt stress had a significant effect on the content of osmotic regulatory substances (Figure 6; *p* < 0.05). The soluble sugar content increased under NaCl stress (Figure 6A) and reached a maximum under 240 mM NaCl treatment, reaching 15.06, 17.53, 18.26, and 13.79%, respectively. After decreasing with increasing NaCl concentration, the soluble protein content (Figure 6B) of all four Magnoliaceae plants generally increased. *Y. denudata* showed the smallest increase, with a maximum value of 43.44 mg·g^−1^ in the 120 mM NaCl treatment, whereas in the others, the maximum soluble protein content reached above 70 mg·g^−1^. After decreasing with increasing NaCl concentration, the proline content (Figure 6C) of Jiaohong 1, *Y. biondii*, and *Y. denudata* increased; in contrast, that of Jiaohong 2 continuously increased. In comparison with those of the other Magnoliaceae plants, Jiaohong 1 had the highest value in the 120–240 mM NaCl treatments, reaching 8.98–9.42 μg·g^−1^.

### 2.5. Effects of NaCl on Ion Contents

**Figure 7:**
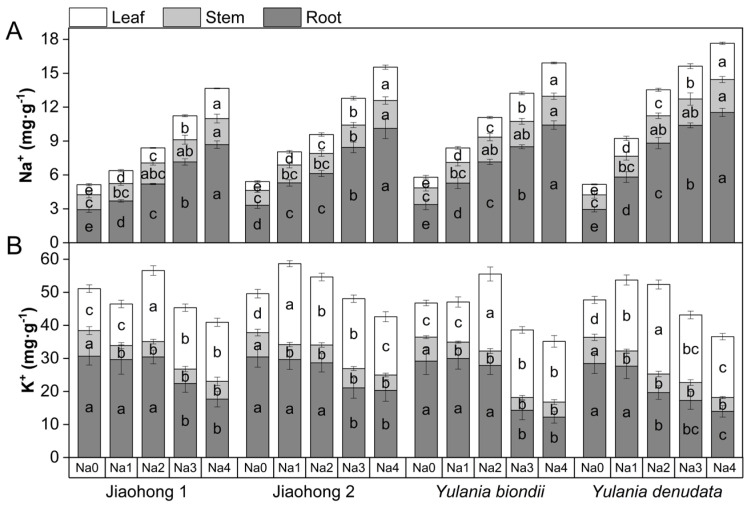
Effects of NaCl on Na^+^ and K^+^ in four Magnoliaceae plants. (**A**) Na^+^; (**B**) K^+^. Na0: 0 mM NaCl; Na1: 60 mM NaCl; Na2: 120 mM NaCl; Na3: 180 mM NaCl; and Na4: 240 mM NaCl. Treatments for a given species were compared. Different lowercase letters in the figure indicate significant differences (*p* < 0.05 by Duncan’s test) among treatments. The bars indicate the standard deviation.

The effects of salt stress on Na^+^ and K^+^ contents are presented in Figure 7. With increasing NaCl concentration, the Na^+^ concentration in the roots, stems, and leaves of the four Magnoliaceae plants increased (Figure 7A). Among them, the Na^+^ content in the roots increased significantly. Among the four Magnoliaceae plants, the Na^+^ contents in the roots, stems, and leaves of *Y. denudata* increased the most, whereas those in Jiaohong 1 increased the least.

K^+^ in the roots of the four Magnoliaceae plants tended to decrease with increasing NaCl concentration (Figure 7B). In *Y. denudata*, K^+^ treatment at 120 mM NaCl and above decreased significantly (*p* < 0.05), whereas in the other three species, there was a significant difference at 180 mM NaCl and above (*p* < 0.05). The K^+^ content in the stems decreased significantly under NaCl stress (*p* < 0.05), and the K^+^ content in the leaves first increased and then decreased with increasing NaCl concentration and was generally higher than that in the control.

### 2.6. Principal Component, Heatmap, and Correlation Analyses for Growth and Physiology Indicators

**Figure 8:**
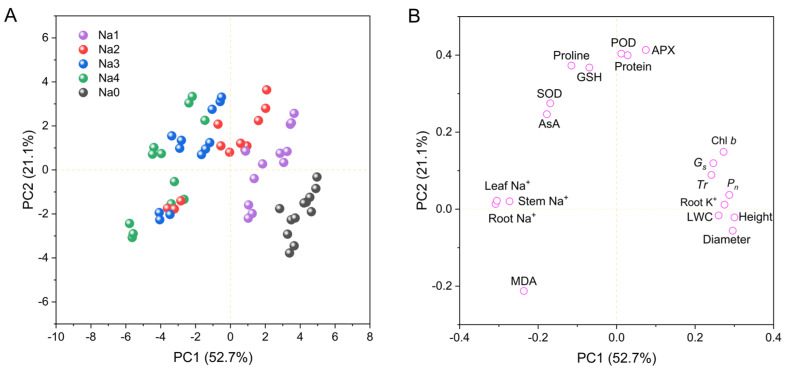
Principal component analysis (PCA) showing the effects of NaCl on growth and phylogenetic indices. (**A**) Score plot; (**B**) Loading plot for PC1 and PC2. PC1, first principal component; PC2, second principal component. Na0: 0 mM NaCl; Na1: 60 mM NaCl; Na2: 120 mM NaCl; Na3: 180 mM NaCl; and Na4: 240 mM NaCl.

Principal component analysis (PCA) was used to analyze the impact of NaCl treatment on growth and physiological indicators (Figure 8). As shown in Figure 8, the factors can be divided into two principal components with contributions of 52.7% and 21.1% to the total variance. A PCA score plot (Figure 8A) revealed obvious differences between the control and NaCl stress treatments, which explained the contribution of the 0–120 mM NaCl treatments to principal component 1 (PC1), whereas the 60–240 mM NaCl treatments clearly contributed to principal component 2 (PC2). The loading plot in Figure 8B shows that growth indicators, including plant height, stem diameter, LWC, Chl *b*, *P_n_*, *G_s_*, *Tr* and root K^+^, contributed the most to PC1, whereas antioxidant and osmotic adjustment indicators, including SOD, POD, APX, GSH, AsA, protein and proline, contributed the most to PC2. However, MDA had a negative contribution to PC1 and PC2, and root Na^+^, leaf Na^+^, and stem Na^+^ had negative contributions to PC1.

**Figure 9:**
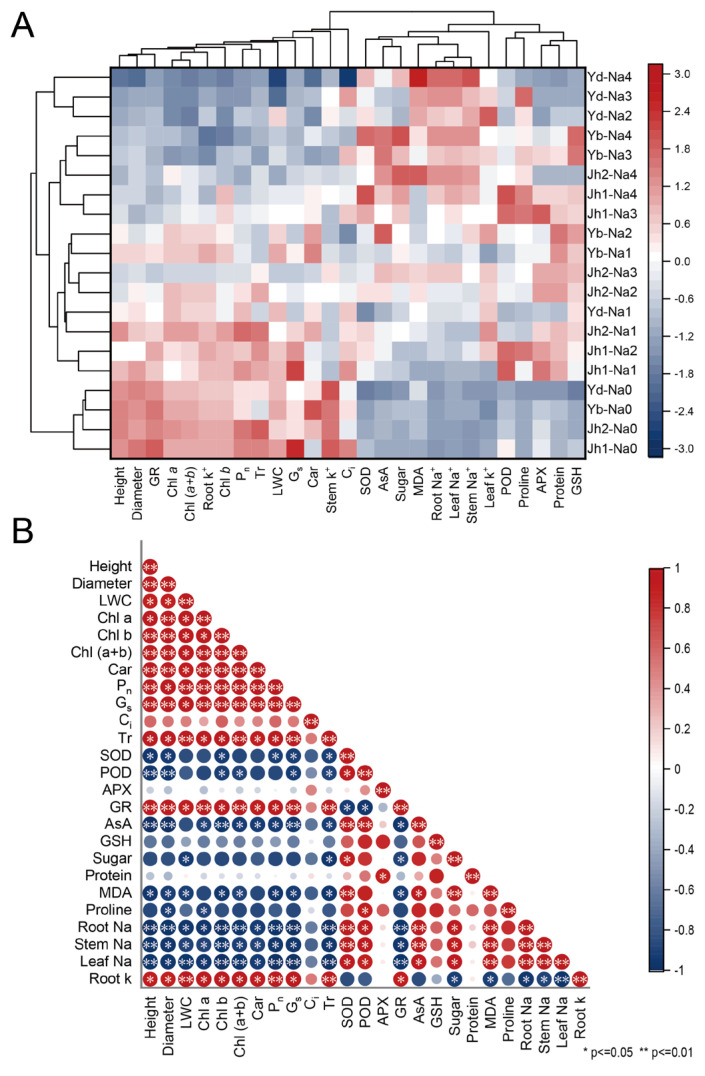
NaCl-induced variation in growth and physiological indicators was analyzed via heatmap analysis and correlation analysis. (**A**) Heatmap analysis; (**B**) Correlation analysis. Jh1: Jiaohong 1; Jh2: Jiaohong 2; Yb: *Y. biondii*; Yd: *Y. denudata*. Na0: 0 mM NaCl; Na1: 60 mM NaCl; Na2: 120 mM NaCl; Na3: 180 mM NaCl; and Na4: 240 mM NaCl.

A heatmap analysis clearly showed the differences in each index between the treatments (Figure 9A). The plants from the twenty treatments were grouped into three groups using clustering analysis. One group of *Y. denudata* plants was subjected to 120–240 mM NaCl treatment; these plants exhibited a low growth rate, low antioxidant activity, high MDA content, and high Na^+^ content. The other group included four Magnoliaceae plants in the 0 mM NaCl treatment; these plants exhibited high growth rates, low antioxidant activities, and low contents of osmotic regulatory substances. The remaining treatments included a group that exhibited a moderate growth rate, high antioxidant activity, and high osmotic regulation substance content.

Correlations among indicators were analyzed by plotting the correlation matrix (Figure 9B). There were significant positive correlations among most growth indicators, including a high relative growth rate, diameter relative growth rate, photosynthetic pigments, *P_n_*, *G_s_*, and *Tr*. There were positive correlations among most antioxidant indicators, including SOD, POD, APX, AsA, and GSH. In contrast, little correlation was detected among the osmotic regulatory substances, and antioxidant activity and osmotic regulatory substances, especially AsA, SOD, and POD, were negatively related to growth indicators. The correlation between antioxidant activity and osmotic regulation substance content was not obvious.

## 3. Discussion

The response of trees to NaCl stress varies greatly among species and cultivars [2,24]. Previous research has suggested that Magnoliaceae plants are typical glycophytes [25]. However, the NaCl tolerance of *M. wufengensis*, a newly discovered species of Magnoliaceae, and how it differs from that of other Magnoliaceae species remains unknown. In this study, the NaCl tolerance of two varieties of *M. wufengensis* (Jiaohong 1 and Jiaohong 2) and *Y. biondii* and *Y. denudata* were compared by determining their morphological and physiological indices, and the main physiological mechanisms of NaCl tolerance were explained. The following sections address this issue in greater detail.

### 3.1. NaCl Treatments Inhibited Plant Growth

The difference in turgor pressure between the inside and outside of cells is due to the water loss caused by the difference, which may be correlated with the capacity of cells to absorb water or the ability of stomata to retain water, and is what causes a decrease in leaf water content (LWC) under NaCl stress [26]. In the present study, NaCl decreased LWC, indicating a decreased capacity for water absorption. The LWC of *Y. denudata* declined more than that of the other species, indicating a decreased capacity for water absorption. Plant height and stem diameter are typical effective indicators used to assess plant growth under NaCl stress. Studies on *Salix eriocephala* [24], *Malus pumila* [27], *Olea europaea* [28], and other woody plants have demonstrated that NaCl stress reduces plant growth. The results of this study are consistent with the finding that NaCl stress reduced the growth rates of plant height and stem diameter in the four Magnoliaceae plants. The growth rates decreased as the NaCl concentration increased, which was related to the osmotic stress induced by NaCl [14]. Increasing soil salinity due to a shortage of water and shrinkage of cells inhibits cell elongation and reduces cell division, thereby reducing the plant growth rate [29,30]. Osmotic stress normally occurs within the first several hours or days of NaCl stress. Plant growth gradually recovers due to osmosis regulation when NaCl is minimal, whereas at medium or high concentrations of NaCl, cell shrinkage caused by osmosis cannot be recovered. Within a few weeks, the inhibitory effect of NaCl on plant growth becomes obvious, leading to a reduced growth rate, reduced leaf area, increased leaf thickness, etc. [14]. The specific degree of inhibition varies among species. Osmotic stress is a nonspecific stress type that occurs in response to many abiotic stresses, such as drought stress [31]. Additionally, the specific ion Na^+^ generated by NaCl stress—known as ion stress—can cause slower growth. At high concentrations, Na^+^ is toxic after it enters cells, leading to protein degradation, membrane lipid peroxidation, and disruption of cellular homeostasis. As a result, the growth and division of plant cells are affected [3]. Ion stress, a specific stress caused by salt, generally occurs in the latter stages of NaCl stress [32]. There were variations among the four Magnoliaceae plants examined in this study in terms of their levels of tolerance to salt stress. *Y. denudata* had a much lower seedling height and stem diameter than the others, which suggested that the plant was less tolerant of NaCl stress.

### 3.2. Photosynthetic Pigment Contents and Photosynthetic Parameter Observations Revealed the Effects of NaCl on Photosynthesis

Chlorophyll and carotenoids are pigments contained in the chloroplasts of higher plants, and variations in their contents are key indicators of plant growth and NaCl tolerance. Chlorophyll may degrade because of free radicals generated by NaCl stress and interference from Na^+^ [33,34]. According to earlier studies, the chlorophyll content of salt-tolerant cultivars increased, whereas that of salt-sensitive cultivars decreased [33]. In the present study, the chlorophyll *a* content of the four plants showed a decreasing tendency as the NaCl concentration increased. The chlorophyll *b* content of Jiaohong 1 showed no obvious change, whereas that of the others showed a decreasing trend. The chlorophyll content of *Y. biondii* and *Y. denudata* decreased more obviously than that of *M. wufengensis*, which may lead to a higher NaCl tolerance in *M. wufengensis*. Carotenoids not only absorb light energy but also act as antioxidants. It has been discovered that carotenoid pigments, such as β-carotene, act as ^1^O_2_ quenchers and protect photosynthetic reaction centers and antennae [35]. In the present study, the content of carotenoids in Jiaohong 1 was not affected by NaCl stress but decreased with increasing NaCl concentration, suggesting that Jiaohong 1 could not only absorb more light energy under stress but also transform and utilize light energy more effectively.

Numerous studies have demonstrated that NaCl can prevent plants from undergoing photosynthesis. According to the findings of Frosi et al. [36], NaCl decreased the photosynthetic attributes of *Cenostigma pyramidale*, including *P_n_*, *G_s_*, and light energy conversion efficiency (*F_v_’*/*F_m_*). In compliance with these findings, earlier reports suggested that *P_n_*, *C_i_*, *G_s_*, and PSII activity, as well as photosynthetic products, including soluble sugars and starches, were adversely affected by NaCl stress in *Linum usitatissimum* [10]. With an increase in NaCl concentration, we observed the same effects on Magnoliaceae plants, where *P_n_*, *G_s_*, and *Tr* decreased under NaCl stress. However, *C_i_* did not obviously change, which differed from the findings of the aforementioned studies. This result was similar to that of *Lilium longiflorum*, where *C_i_* under NaCl stress increased or remained unaltered [2]. The *C_i_* of *Persea americana* increased with increasing NaCl concentration and was accompanied by decreases in the *P_n_*, *G_s_*, *Tr*, and chlorophyll contents [37]. The reduction in the photosynthetic rate under NaCl stress is mainly caused by stomatal and nonstomatal factors. Stomatal closure (stomatal limitation) and a reduction in CO_2_ absorption during the early stages of NaCl stress are the main factors causing a decrease in photosynthesis [38]. In the early stage or at a low degree of NaCl stress, *P_n_* is thought to decline mostly due to stomatal factors and can be restored by adjusting the plant’s osmotic balance or opening stomata [38]. Nonstomatal factors include disruption of chloroplast function, interference with CO_2_-fixing enzymes, and disruption of the thylakoid membrane via proton-motive forces caused by ion stress [32]. Previous studies on *Salix eriocephala* have shown that *C_i_* decreases while chlorophyll fluorescence parameters remain unchanged, demonstrating that PSII is not damaged by NaCl; thus, it is speculated that the primary reason for the decrease in *P_n_* was stomatal factors [24]. At the late stage or when plants are under a high level of NaCl stress, when the structural integrity of the photosystem is irreparably damaged, the decrease in *P_n_* may be influenced by both stomatal and nonstomatal factors [26]. The variation in *C_i_* in this study indicated that the photosynthesis of Magnoliaceae was affected by stomatal and nonstomatal factors after 60 days of NaCl treatment, demonstrating greater suppression of CO_2_ fixation or additional damage to the photosynthetic apparatus.

### 3.3. Antioxidant Enzymes and Nonenzymatic Antioxidants Work Together

ROS production in chloroplasts, mitochondria, cell membranes, and nuclei is a normal response in plants under normal conditions. A modest amount of ROS contributes to signaling processes under both biotic and abiotic stresses, but excessive ROS may result in oxidative damage to plants [7]. Insufficient energy use during the photosynthetic process under NaCl stress is the primary reason for ROS overproduction [6]. Excessive ROS production leads to not only the destruction and functional alteration of carbohydrates, proteins, and nucleic acids in cells but also membrane lipid peroxidation and electrolyte leakage [7,39]. MDA, the final decomposition product of membrane lipid peroxidation, is used to gauge the severity of stress in plants [40]. Although the MDA content varies among various plants, it increases when plants are exposed to NaCl stress [33,40]. Following NaCl stress, the MDA content of Magnoliaceae plants significantly increased, indicating an increase in ROS and the development of membrane lipid peroxidation. Sarker and Oba [33] reported more ROS and membrane lipid peroxidation in salt-sensitive varieties of *Amaranthus tricolor* than in salt-tolerant varieties. Jiaohong 1 had much less MDA than the other Magnoliaceae plants, which may have been due to an improved antioxidant system.

Plants utilize both antioxidant enzymes and nonenzymatic antioxidants to scavenge ROS. The first stage in ROS decomposition is the conversion of O_2_^•−^ by SOD to H_2_O_2_, after which H_2_O_2_ is converted to H_2_O by CAT, APX, etc. [7]. As observed in our study, the increase in SOD activity under salt stress may be due to increased O_2_^•−^, which is consistent with the findings of several previous studies [40]. However, several studies have shown that as NaCl concentration increases, SOD activity first increases and then decreases [41]. A decrease in SOD activity after NaCl treatment has also been reported [6]. This indicates that there may be complex interactions between antioxidant enzyme activity and salt tolerance that may be related to the cultivar or NaCl concentration.

The redox balance of plants must be maintained through the AsA–GSH cycle. AsA converts hydrogen peroxide into water in the presence of APX [7]. A previous study revealed that APX was more efficient than CAT in the decomposition of ROS [33]. MDHA, one of its products, unstably decomposes into AsA and DHA. DHA and GSH subsequently interact to create AsA. GR catalyzes the production of GSH [7]. In the present study, with increasing NaCl concentration, the APX activity first increased and subsequently decreased, possibly resulting from increased Na^+^ at high salinity, which inhibited the enzyme activity. However, the AsA content increased with increasing NaCl concentration, probably because at low salinity, plants sped up the AsA–GSH cycle to remove excessive ROS, whereas at high NaCl stress, the decline in APX activity slowed AsA consumption. A decrease in GR activity with increasing NaCl concentration may cause the inhibition of enzyme activity by Na^+^, which also limits the participation of GSH in this cycle. The GSH content in Jiaohong 1 and *Y. biondii* increased, whereas that in Jiaohong 2 and *Y. denudata* first increased and then decreased. Furthermore, *Y. denudata* had the lowest APX activity, which may be related to its varying NaCl tolerance, and it had a decreased capacity to remove ROS via the AsA–GSH cycle.

### 3.4. Osmotic Regulatory Substances Used to Resist NaCl Stress

To resist the decrease in osmotic pressure caused by NaCl stress, plants produce highly water-soluble and nontoxic high-concentration osmotic substances, such as soluble sugar, soluble protein, and proline [42,43]. These results suggest that these osmotic regulatory substances could effectively regulate osmotic pressure. Soluble sugars in plants perform various functions, serving as building blocks for various osmolytes in addition to serving as osmotic protectants and substrates for energy production [44,45]. Under NaCl stress, soluble sugar content has been found to increase in numerous studies [36,42]. This result showed that the soluble sugar content increased as the NaCl concentration increased, but the soluble sugar content in *Y. denudata* was much lower than that in the other Magnoliaceae plants. In the present study, the soluble protein content first increased and subsequently decreased with increasing NaCl concentration. This might be because plants synthesize soluble proteins to resist osmotic stress at low salinity, but at high salinity, ion toxicity leads to a large amount of protein destruction, which lowers the quantity of soluble proteins. The manner in which the four Magnoliaceae plants react to soluble proteins varies. In general, *Y. denudata* increased the least, indicating that it has the lowest capacity to produce soluble proteins.

Proline is the main osmotic substance synthesized by plants under stress and is crucial for protecting membranes, maintaining ionic homeostasis, and scavenging active oxygen [42]. In the present study, the proline content varied among different Magnoliaceae plants. The proline content of *M. wufengensis* increased with increasing salt concentration, whereas that of *Y. biondii* and *Y. denudata* first increased and then decreased. Under high salt concentrations, proline accumulation in Jiaohong 1 was significantly higher than that in the other plants. A higher proline content leads to better adaptation to NaCl stress [26]. The stronger osmotic protection function of Jiaohong 1’s organic osmotic adjustment chemicals could be partly responsible for its superior adaptability to NaCl stress compared to that of the other plants. *Y. denudata* has a low level of organic osmotic adjustment chemicals, which contributes to its poor NaCl tolerance.

### 3.5. Na^+^ and K^+^ Contents Changed under NaCl Stress

The absorption and transport of Na^+^, the main harmful ion to plants under NaCl stress, have always been the focus of research on salt stress. The capacity of a plant to limit Na^+^ absorption, expel Na^+^ from the root system, and prevent Na^+^ from traveling to aboveground components is related to its ability to resist NaCl [46,47]. This study showed that the roots of Magnoliaceae could intercept Na^+^ because the concentration of Na^+^ was greater in roots under NaCl stress than in stems and leaves. However, there was some variation among the four Magnoliaceae plants. Compared to the other three Magnoliaceae plants, *Y. denudata* exhibited a greatly reduced ability to prevent Na^+^ absorption. Because Na^+^ has a competitive inhibitory impact on K^+^ absorption, the high levels of Na^+^ that enter cells under NaCl stress affect K^+^ absorption [48]. In the present study, the K^+^ content in Magnoliaceae tended to increase first and then decrease, which is among the NaCl tolerance strategies. Plants block the entry of Na^+^ into root cells at low NaCl concentrations by absorbing an excessive amount of K^+^. However, during severe NaCl stress, a significant amount of Na^+^ enters cells because the plant membrane system breaks down, which lowers the K^+^ level [46,49].

### 3.6. Principal Component, Heatmap, and Correlation Analyses Reveal the Salt Tolerance Mechanism

Principal component analysis (PCA) is a widely used statistical method that converts multiple indices into a smaller number of comprehensive indices to reveal their relationships. In this study, multiple indices were divided into two principal components: growth indicators and osmotic antioxidant indices (Figure 8B), which was consistent with the findings of the cluster analysis (Figure 9A). These findings reveal a conflict between plant growth and resistance to salt stress. Under normal conditions, plants use energy from photosynthesis to produce carbohydrates for growth, whereas under salt stress, plants must sacrifice a part of their growth to produce antioxidants and osmoregulatory substances, which help them resist salt stress [14,32]. This is a survival strategy used by plants under salt stress. In the present study, correlation analysis (Figure 9B) revealed a negative relationship between growth indices and antioxidant osmotic regulatory indices, which supports this conclusion. The associations between the five treatments and the principal components were determined using a score plot (Figure 8A). Energy was primarily used for growth under a low NaCl concentration (60 mM) or control. With an increase in salt concentration, less energy was used for growth, and more energy was used for osmotic and antioxidant regulation. At NaCl concentrations of 180–240 mM, the PC1 score was negative, indicating that plant growth was hindered.

Differences in salt tolerance may be observed among different species of trees or even cultivars, which have been examined in various trees [24,28]. Differences in salt stress tolerance between species can be determined through heatmap analysis and cluster analysis (Figure 9). According to the growth indices at low salt concentrations, Magnoliaceae were not affected or were less affected by salt stress. In addition, Magnoliaceae exhibited moderate growth while producing more antioxidants and osmoregulatory substances under moderate salinity, indicating that the plants expended some energy synthesizing these substances to resist salt stress. Jiaohong 1 had the highest levels of osmoregulatory substances and antioxidants, indicating that it has a high capacity to withstand salt stress. At high salinity, the plants exhibited poor growth and antioxidant and osmotic indices, indicating poor growth conditions. Furthermore, membrane damage and protein destruction caused by ion stress inhibit plant growth and prevent the synthesis of antioxidant and osmotic substances, which may lead to cell death and even plant death [32]. Increased soil salinity leads to a lack of water and cell shrinkage. At high salinity, the growth and photosynthesis capacity of *Y. denudata* were the lowest, and there were insufficient osmotic and antioxidant substances, indicating that *Y. denudata* has a lower salinity tolerance than the other species.

The tolerance range of NaCl and physiological mechanisms were investigated in the present study of NaCl tolerance in four Magnoliaceae plants, which is highly important for exploring the underlying molecular mechanisms and methods used to improve salt tolerance. This research is also of great practical significance for the transplantation and popularization of Magnoliaceae plants in coastal and other saline areas. However, this study considered only the effects of NaCl, and the effects of combined salt stress on Magnoliaceae plants should be considered in the future. 

## 4. Materials and Methods

### 4.1. Plant Materials and Growth Conditions

For this experiment, *Yulania biondii* seedlings, *Yulania denudata* seedlings, and two varieties of *M. wufengensis* grafted seedlings (Jiaohong 1 and Jiaohong 2) were used. Two-year-old *Y. biondii* and *Y. denudata* plants were kindly provided by Wufeng Bo Ling Seed Industry Co., Ltd., from Wufeng, China, and transplanted in November 2020. Jiaohong 1 and Jiaohong 2 plants were grafted in March 2021 using *Y. biondii* as the rootstock. The experiment was conducted from June to September 2021 in Beijing, China (40°3′54″ N, 116°05′45″ E), in a greenhouse with a controlled temperature that averaged 22 °C during the day and 18 °C at night. The experiment was conducted in nondraining plastic pots (360 mm in diameter and 320 mm in height) with one plant and 3.5 kg of turfy soil in each pot.

### 4.2. Experimental Design

A randomized complete block design of two factors (four Magnoliaceae plants × five NaCl concentrations) was used in this study. Each treatment consisted of 10 healthy seedlings for a total of 20 treatments. The settings of the NaCl concentrations are shown in Table 1. NaCl was applied in the form of a salt solution. to avoid seedling death when the concentration was too high, the salt solution was divided four times on average, with an interval of 5 days for each time, until the treatment was completed on the 20th day. In the experiment, the water loss from the potted soil was weighed by the weighing method and supplemented with water every day, and the soil moisture content in the potted soil was kept at 70~80% of the field water capacity. The growth conditions and salt stress symptoms of the plants were observed every 15 days. Plant height, stem diameter, leaf water content, and photosynthetic parameters were measured on the 60th day, and the mature leaves in the middle and upper parts were quickly frozen with liquid nitrogen and then placed in a freezer at −80 °C to determine physiological indices. Three plants were randomly selected from each treatment, and samples were obtained from the roots, stems, and leaves to determine the ion content.

### 4.3. Plant Growth

**Figure 10:**
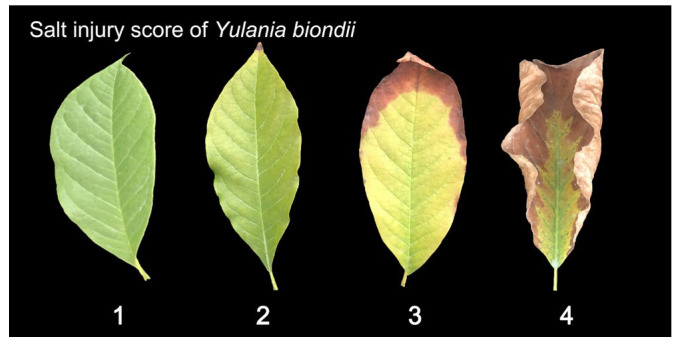
Salt injury score of four species (using *Yulania biondii* as an example).

The growth conditions and salt stress symptoms of the plants were observed every 15 days. The standard was classified according to the methods of Niu et al. [47], with some modifications. The salt injury score was assessed using a 1–4 score as follows (Figure 10):

1: Healthy leaves; 

2: Slightly yellow or damaged leaves; 

3: Moderately yellowed and scorched leaves; 

4: Severely scorched and fallen leaves.

The salt injury index (SI) was calculated as follows [50]:

(1) $$SI\;\left(\%\right)=\frac{\sum \;\left(s\times n\right)}{N\times S}\times 100$$

where: s = the value of each score, n = the number of plants in each grade, N = the total number of plants, and S = the highest injury score.

The leaf water content (LWC) was calculated as follows:

(2) $$LWC(\%)=\frac{fresh\;weight-dry\;weight}{fresh\;weight}\times 100$$

Plant height and stem diameter were measured individually at 0 d and 60 d to calculate the relative growth rate (%).

### 4.4. Photosynthetic Pigment Contents and Photosynthetic Parameters

The concentrations of chlorophyll *a* (mg·g^−1^), chlorophyll *b* (mg·g^−1^) and carotenoids (mg·g^−1^) were quantified using the spectrophotometric method described by Lichtenthaler and Wellburn [51] using 0.20 g of fresh ground leaves. The samples were stored in 10 mL of 95% ethanol for 24 h, after which the absorbance was measured at 665, 649, and 470 nm using a spectrophotometer (VERTEX70, Bruker, Billerica, MA, USA).

The fully grown leaves at the middle and upper parts were used to evaluate the photosynthetic rate (*P_n_*, μmol·m^−2^·s^−1^), stomatal conductance (*G_s_*, mmol·m^−2^·s^−1^), intercellular CO_2_ concentration (*C_i_*, μmol·m^−2^·s^−1^), and transpiration rate (*Tr*, μmol·m^−2^·s^−1^) attributes using a portable infrared gas analyzer (IRGA) photosynthetic system (LI-COR 6400, LI-COR, Lincoln, NE, USA) between 9:00 and 11:00 h, with open-air flow and an internal PAR of 1000 μmol·m^−2^·s^−1^.

### 4.5. Antioxidant Enzyme Activities, Nonenzymatic Antioxidants, and Membrane Lipid Peroxidation

After fresh leaves (0.1 g) were ground in 5 mL of 50 mmol·L^−1^ phosphate buffer (PBS, pH = 7.8) and centrifuged at 10,000× *g* for 20 min (4 °C), the supernatant was collected for use in the enzyme assay. 

Superoxide dismutase (SOD, U·g^−1^) was assayed using nitroblue tetrazolium [52]. The enzyme extract (50 μL) was mixed with 1.5 mL of PBS (0.05 mol·L^−1^, pH 7.8), 0.3 mL of L-Met (130 mmol·L^−1^), 0.3 mL of NBT (750 μmol·L^−1^), 0.3 mL of EDTA-Na_2_ (100 μmol·L^−1^) and 0.3 mL of riboflavin (20 μmol·L^−1^). One unit of SOD activity (U·g^−1^) was defined as the amount of enzyme needed to cause 50% inhibition of NBT reduction, as measured at 560 nm.

Peroxidase (POD, U·g^−1^) activity was determined following the guaiacol method [52]. The enzyme extract (100 μL) was added to a mixture of 2.9 mL of PBS (50 mmol·L^−1^, pH 7.0), 1 mL of H_2_O_2_ (12%), and 1 mL of guaiacol (50 mmol·L^−1^). Trichloroacetic acid was added after 15 min in a 37 °C water bath to stop the reaction. The absorbance value was recorded at 470 nm.

Ascorbate peroxidase (APX, μmol·g^−1^·min^−1^) activity was measured as described by Kamran et al. [52]. The enzyme extract (100 μL) was added to the APX reaction mixture containing PBS (50 mmol·L^−1^, pH 7.0), 0.1 mL of ascorbate (5 mmol·L^−1^) and 0.1 mL of H_2_O_2_ (20 mol·L^−1^). The change in absorbance due to ascorbate consumption was monitored at 290 nm.

The activity of glutathione reductase (GR, U·g^−1^) was determined following the procedures of Hossain [53]. The reaction mixture contained 1.7 mL of PBS (50 mmol·L^−1^, pH 7.8), 0.1 mL of GSSG (10 mmol·L^−1^) and 0.1 mL of NADPH (2.4 mmol·L^−1^). The decrease in absorbance at 340 nm due to NADPH oxidation was recorded for 1 min. 

Ascorbic acid (AsA, μg·g^−1^) was measured using the procedure described by Wang et al. [54] with some modifications. Ground leaves (0.5 g) were centrifuged (10,000× *g*) for 10 min (4 °C) with 5 mL of TCA (6%). Two milliliters of the supernatant were mixed with 0.6 mL of PBS (200 mmol·L^−1^, pH 7.4), 0.2 mL of distilled water, 0.8 mL of H_3_PO_4_ (42%), 0.8 mL of 2,2′-bipyridyl (2%), and 0.4 mL of FeCl_3_ (3%), after which the mixture was heated in a 42 °C water bath for 50 min. The absorbance of the supernatant was recorded at 525 nm.

Reduced glutathione (GSH, μmol·g^−1^) was measured using the procedure described by Wang et al. [54] with some modifications. Fresh samples (0.1 g) were extracted with 1.5 mL of 5% sulfosalicylic acid. The homogenates were centrifuged at 14,000× *g* for 10 min (4 °C), and the resulting extracts were subjected to enzyme assays. Then, 0.2 mL of the supernatant was mixed with 1.4 mL of PBS (50 mmol·L^−1^), 0.1 mL of DTNB (15 mmol·L^−1^), 50 μL of NADPH (10 mmol·L^−1^) and 30 μL of GR (50 μmol·L^−1^). The reaction was initiated by the addition of glutathione reductase, and the increase in absorbance at 412 nm was monitored.

Lipid peroxidation was determined by estimating the malondialdehyde (MDA, μmol·g^−1^) concentration using the thiobarbituric acid (TBA) method [52]. Ground leaves (0.5 g) were centrifuged (12,000× *g*) for 25 min (4 °C) with 5 mL of TCA (5%). Two milliliters of the supernatant were mixed with 2 mL of TBA, heated in a boiling water bath for 30 min, cooled in an ice bath, and centrifuged again (10,000× *g*) for 5 min. The absorbance of the supernatant was recorded at 450, 532, and 600 nm.

### 4.6. Estimation of Osmotic Adjustment

The soluble sugar content (%) was determined using the anthrone–sulfuric acid method [52]. A 0.1 g sample was extracted with 5 mL of water at 100 °C for 30 min, followed by filtration and dilution with water to 50 mL. The extract was mixed with 1.5 mL of water, 0.5 mL of anthrone, and 5 mL of concentrated sulfuric acid. The absorption of each sample was measured at 630 nm.

The soluble protein content (mg·g^−1^) was measured as described by Bradford [55]. Fresh leaf samples (0.1 g) were homogenized in 5 mL of PBS (50 mmol·L^−1^, pH 7.8). Homogenization (0.1 mL) was followed by the addition of 0.9 mL of distilled water and 5 mL of Coomassie Brilliant Blue G250. The absorbance was subsequently read at 595 nm.

The proline content (μg·g^−1^) in the leaves was estimated using the method proposed by Ringel et al. [56]. A 0.5 g sample was extracted with 5 mL of sulfosalicylic acid (3%) at 100 °C for 30 min, followed by filtration. Two milliliters of the extract was mixed with 2 mL of glacial acetic acid and 2 mL of ninhydrin, heated in a boiling water bath for 30 min, cooled in an ice bath, and added to 4 mL of methylbenzene. The mixture was shaken, and the supernatant was centrifuged at 3000× *g* for 5 min. The absorbance was recorded at 520 nm.

### 4.7. Ion Contents

Three plants were randomly selected from each treatment, and their roots, stems, and leaves were collected for ion content determination. The roots, stems, and leaves were separated, washed, dried, and then placed in an oven at 105 °C for 1 h and 60 °C for 48 h. The dried sample was ground into powder using a ball mill (MM 400, Retsch, haan, Germany) and then digested with H_2_SO_4_ and H_2_O_2_. The contents of Na^+^ (mg·g^−1^) and K^+^ (mg·g^−1^) were determined via atomic absorption spectrophotometry (TAS-990AFG, Persee, Beijing, China).

### 4.8. Statistical Analysis

All statistical analyses were performed using SPSS Statistics (version 22.0, IBM, Armonk, NY, USA). The data are presented as mean ± standard deviation (SD) from three independent experiments, and significant differences were determined using two-way ANOVA followed by Duncan’s multiple comparisons test. The ANOVA results are presented in the Supplementary Materials. Differences between the two indicated settings were considered statistically significant at *p* < 0.05. Graphs were constructed using Origin Pro (version 2021, Origin Lab, Northampton, MA, USA).

## 5. Conclusions

In summary, the work presented in this study shows the growth and physiological changes in four Magnoliaceae plants under NaCl stress and reveals the potential mechanism of NaCl tolerance through principal component analysis, cluster analysis, and correlation analysis. According to these findings, NaCl treatment caused Na^+^ accumulation, reduced photosynthetic pigment contents and photosynthetic rates, damaged the membrane system, interfered with K^+^ absorption, and inhibited plant growth while increasing the antioxidant and osmoregulatory substance content. In the present study, the salt-sensitive and salt-tolerant plants behaved differently under NaCl stress. *Y. denudata* exhibited decreased growth and photosynthesis, as well as a decreased ability to synthesize antioxidants and osmotic adjustment substances, indicating that its NaCl tolerance was lower than that of the other three Magnoliaceae plants. In general, the order of salinity tolerance of the four Magnoliaceae plants was Jiaohong 1 > Jiaohong 2 > *Yulania biondii* > *Yulania denudata*. This study revealed the physiological mechanism of NaCl tolerance in Magnoliaceae and provided a basis for the planting and spreading of Magnoliaceae in saline areas.

## Figures and Tables

**Table 1:** NaCl solution addition amount for each treatment group.

Treatment	Soil NaCl Content (Weight Ratio)	Soil Electric Conductivity (ms·cm^−1^)	Soil Weight (kg)	NaCl Weight (g)	NaCl Solution Concentration (mM)	Total NaCl Solution Volume (L)
Na0	0‰	0.213	3.5	0	0	1
Na1	1‰	0.421	3.5	3.5	60	1
Na2	2‰	0.784	3.5	7.0	120	1
Na3	3‰	1.592	3.5	10.5	180	1
Na4	4‰	3.021	3.5	14.0	240	1

## Data Availability

Data will be made available on request.

## Supplementary Materials

The following supporting information can be downloaded at: https://www.mdpi.com/article/10.3390/plants13020170/s1, Table S1: *p* Value and significant levels (Two-way ANOWA) of growth and physiological indices in four Magnoliaceae plants under NaCl stress.
