# Supplementary material for: Growth and Physiological Responses of Magnoliaceae to NaCl Stress
Source: Plants (Basel). 2024 Jan 8;13(2):170. doi: 10.3390/plants13020170 (PMC10818768; doi:10.3390/plants13020170)
Supplement: Supplementary file 1 [file plants-13-00170-s001.zip › plants-2747182-supplementary.pdf]

Table S1 *P* Value and significant levels (Two-way ANOWA) of growth and physiological indices in four Magnoliaceae plants under NaCl stress.

Indices	Plant	NaCl level	Interaction
Leaf water content	<0.000 1***	<0.000 1***	0.009**
Relative growth rate of plant height	<0.000 1***	<0.000 1***	<0.000 1***
Relative growth rate of stem diameter	<0.000 1***	<0.000 1***	<0.000 1***
Chlorophyll *a*	<0.000 1***	<0.000 1***	<0.000 1***
Chlorophyll *b*	<0.000 1***	<0.000 1***	<0.000 1***
Carotenoid	<0.000 1***	<0.000 1***	<0.000 1***
P_n	<0.000 1***	<0.000 1***	<0.000 1***
G_s	<0.000 1***	<0.000 1***	<0.000 1***
C_i	0.002**	<0.000 1***	0.014*
Tr	<0.000 1***	<0.000 1***	0.003**
SOD	0.015*	<0.000 1***	0.102
POD	<0.000 1***	<0.000 1***	<0.000 1***
APX	<0.000 1***	<0.000 1***	0.009**
GR	0.022*	<0.000 1***	0.840
AsA	<0.000 1***	<0.000 1***	0.077
GSH	<0.000 1***	<0.000 1***	<0.000 1***
MDA	<0.000 1***	<0.000 1***	<0.000 1***
Soluble sugar	<0.000 1***	<0.000 1***	0.664
Soluble protein	<0.000 1***	<0.000 1***	0.005**
Proline	0.001**	0.036*	0.040*
Root Na^+	<0.000 1***	<0.000 1***	<0.000 1***
Stem Na^+	0.004**	<0.000 1***	0.553
Leaf Na^+	<0.000 1***	<0.000 1***	<0.000 1***
Root K^+	<0.000 1***	<0.000 1***	0.051
Stem K^+	0.183	<0.000 1***	0.137
Leaf K^+	<0.000 1***	<0.000 1***	<0.000 1***

*, **, *** significance at $P < 0.05$, 0.01, and 0.001, respectively.
